# Mini Review: Circular RNAs as Potential Clinical Biomarkers for Disorders in the Central Nervous System

**DOI:** 10.3389/fgene.2016.00053

**Published:** 2016-04-06

**Authors:** Dan Lu, An-Ding Xu

**Affiliations:** Department of Neurology and Stroke Center, The First Affiliated Hospital, Jinan University GuangzhouGuangdong, China

**Keywords:** circular RNAs, central nervous system, biomarker, exosome, non-coding RNAs

## Abstract

Circular RNAs (circRNAs) are a type of non-coding RNAs (ncRNAs), produced in eukaryotic cells during post-transcriptional processes. They are more stable than linear RNAs, and possess spatio-temporal properties. CircRNAs do not distribute equally in the neuronal compartments in the brain, but largely enriched in the synapses. These ncRNA species can be used as potential clinical biomarkers in complex disorders of the central nervous system (CNS), which is supported by recent findings. For example, ciRS-7 was found to be a natural microRNAs sponge for miRNA-7 and regulate Parkinson’s disease/Alzheimer’s disease-related genes; circPAIP2 is an intron-retaining circRNA which upregulates memory-related parental genes PAIP2 to affect memory development through PABP reactivation. The quantity of circRNAs carry important messages, either when they are inside the cells, or in circulation, or in exosomes released from synaptoneurosomes and endothelial. In addition, small molecules such as microRNAs and microvesicles can pass through the blood–brain barrier (BBB) and get into blood. For clinical applications, the study population needs to be phenotypically well-defined. CircRNAs may be combined with other biomarkers and imaging tools to improve the diagnostic power.

## Introduction

Circular RNAs (CircRNAs) are a family of naturally occurring endogenous ncRNAs with widespread distribution and diverse functions. They are ∼100 nucleotides long (Memczak et al., 2013) single-stranded RNA molecule forms a circle through covalent binding (Chen et al., 2015), which are highly represented in the eukaryotic transcriptome and abundant in exosomes. A large number of circRNAs have been identified, and some have been validated to function as microRNA sponges in mammal cells through high-throughput RNA sequencing and bioinformatic analysis (Chen et al., 2015; Li Y. et al., 2015). Another important feature of circRNAs is their spatio-temporal specific expression which suggests potential regulatory roles (Shen et al., 2015). In addition, circRNAs have been shown to serve as biomarkers for a potential non-invasive diagnosis for atherosclerosis (Burd et al., 2010), disorders of the central neural diseases (Lukiw, 2013), degenerative diseases (Ashwal-Fluss et al., 2014), and cancers (Li P. et al., 2015; Li Y. et al., 2015).

Finding biomarkers for an accurate diagnosis at an early stage is crucial for the treatment of many complex CNS disorders. For example, oligoclonal bands and IgG ratio is used for prognosis and prediction of conversion from clinically isolated syndrome to multiple sclerosis (MS); JCV Ab seropositive is predictive for progressive multifocal leukoencephalopathy; and MRI is the primary tool for determining phase II and III clinical trials for MS, which has been used in clinic diagnosis and evaluation of prognosis (Housley et al., 2015); the ratios of tau/Aβ42 and p-tau/Aβ42 in the CSF can be used in differential diagnosis, prediction of conversion and the rate of progression from cognitive normalcy to mild dementia and severe impairment in Alzheimer’s disease (AD; Jin et al., 2013); imaging markers including PET and MRI can predict outcome after reperfusion therapies for acute ischemic stroke (Dani and Warach, 2014). However, when patients are diagnosed with Parkinson’s disease (PD), if their dopaminergic neurons have already degenerated by over 60%; the risk of thrombolysis is difficult to assess after patients diagnosed with stroke for 4.5 h (Nathaniel et al., 2015).

CircRNAs are abundant in the brain and exosomes. Their capability to transverse the blood–brain barrier (Li Y. et al., 2015) makes them perfect candidates as potential diagnostic tools for CNS disorders. The circRNAs are enriched by at least twofold in exosomes compared to those retained in the cells (Li Y. et al., 2015), so what could be detected in the human blood may provide information about the disease status in the CNS. Therefore, this mini-review will provide a new approach for biomarker studies in CNS disease area by integrating the circRNAs-related literatures.

### CircRNAs Regulated in CNS Functions and Disorders

Rybak-Wolf et al. (2015) have detected 15,849 circRNA candidates with distinct spatio-temporal expression pattern in mouse and 65,731 in human brain samples. These RNAs are generated from “back-splicing” annotated exons during post-transcriptional processes with a clear preference for coding sequence (CDS) and 5′ untranslated region (UTR) exons (Westholm et al., 2014; Rybak-Wolf et al., 2015). After systematic investigation, unique circRNAs are often differentially expressed in mammalian cells. For example, mouse circRNA generated from Rims2 in the adult brain is expressed 20-fold higher than the linear mRNA, but is lowly expressed in other mouse tissues (Rybak-Wolf et al., 2015); circRims2, circElf2, and circDym are enriched in the cerebellum, while circPlxnd1is enriched in the cortex (Rybak-Wolf et al., 2015); circRNA CiRS-7 is the most highly expressed circRNA in cerebellum of E115 but lowly express in cortex at E60 during porcine embryonic brain development (Veno et al., 2015), which suggests that some circRNAs expression increases during development of the central nervous system (CNS) to augment density of miRNA target sites with respect to the bulk linear coding regions (Westholm et al., 2014). In addition, circRNAs may decrease during cell proliferation in some cancer cells (Bachmayr-Heyda et al., 2015; Chen et al., 2015). A large proportion of circRNAs are abundant in brain, but circRNAs are inequally distributed in the neuronal compartments. with the highest expression in the synapses for brain development or synaptic plasticity. This may explain that fact that synaptic density in the human cerebral cortex may be four times higher than in the mouse brain (Herculano-Houzel, 2009; Veno et al., 2015).

ciRS-7 contains more than 70 selectively conserved miRNA target sites and functions as a miR-7 sponge to regulate the expression of human EGFR, SNCA and IRS2 (Hansen et al., 2013) as well as α-synuclein in PD. If a deficiency occurs in ciRS-7 “sponging” function, miRNA-7 will be released to potentially down-regulate AD- relevant targets, such as the ubiquitin protein ligaseA (UBE2A). UBE-2A is an autophagic, phagocytic protein essential in the clearance of amyloid peptides in AD and other progressive inflammatory degenerations of the human CNS, which is depleted in AD brain (Lukiw, 2013).

CircHomer1a is significantly up-regulated in primary hippocampal neurons. Strikingly, upregulation of circHomer1-a could prevent the potential overexpression of Homer1b/c which may be detrimental to homeostatic synaptic downscaling (You et al., 2015), indicating that circHomer1a could be used as a promising therapeutic target for the treatment of chronic inflammatory pain (Tappe et al., 2006). In addition, downregulation of Homer1b/c could alleviate cytoplasmic calcium levels and neural lactate dehydrogenase release, and ultimately decrease the apoptotic rate after traumatic neuronal injury (Fei et al., 2014). This is supported by the fact that regulating the scaffold protein Homer1 may connect metabotropic glutamate receptors (mGluRs) to the endoplasmic reticulum (Rickhag et al., 2006). CircPAIP2 is an intron-retained circRNAs associated with human RNA polymerase II localized in the nuclei, supporting its potential function in interacting with U1 small nuclei ribonucleoprotein (Li Z. et al., 2015). Therefore, upregulating its parental gene PAIP2 in *cis* may affect translational inhibition of contextual memory-related genes through PABP reactivation (Khoutorsky et al., 2013).

Burd et al. (2010) identified circular ANRIL products emanating from the ANRIL locus and deemed them as causal variants at 9p21.3 to regulate INK4/ARF expression, challenging the notion of atherosclerosis risk in association with modulating ANRIL expression and/or structure (Burd et al., 2010). However, whether circular ANRIL tweaks epigenetic regulation in cardiovascular risk is not clearly understood yet (Perkel, 2013).

CircRNAs possibly serve as a layer of regulation in protein synthesis (Chen and Sarnow, 1995; Hentze and Preiss, 2013). They may bind to mRNA directly to drive translation inside cells, or bind to RNA-binding proteins (RBPs) such as Argonaute, RNA polymerase II and MBL and play a role in the regulation of alternative splicing (Westholm et al., 2014). All these potential mechanisms remain to be further investigated in CNS.

### CircRNAs as Potential Biomarkers for Diagnosis and of Prognosis in CNS Disorders

**FIGURE 1:**
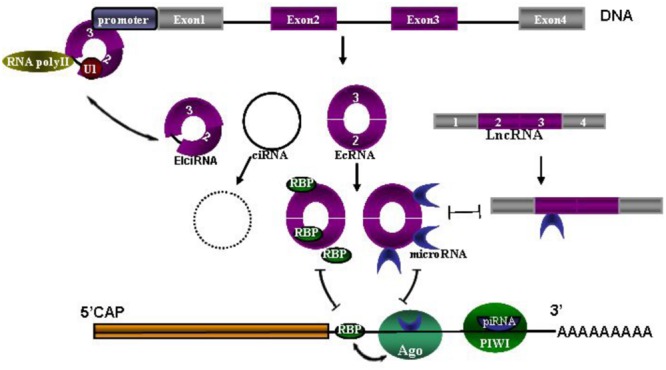
**The network of circRNAs, microRNAs, piRNA and lncRNAs.** Coding sequence (CDS) and 3′ untranslated region (UTR) are followed by polyA tail. MicroRNAs are bound with Argonaute (Ago) proteins and also bound to partially complementary sites in the 3′UTR. Similarly, piRNAs loaded in PIWI associate with the 3′UTR by full complementarity binding. Ago-miRNA activity is further modulated by adjacent RNA-binding proteins (RBPs), presumably by interacting with target proteins. Three types of circRNAs are spliced from DNA, including exon-shuﬄing-derived circRNA (ecRNA), which is only comprised of exons; circular intronic RNA (ciRNA), which is the byproduct formed by intron after pos-transcription; and elciRNAs (exon–intron RNAs), which is consisted of exon and retained intron, and long intron with reverse complementary sequences flanking the joined exons. CiRNAs and elciRNAs can interact with transcription machinery (RNA Pol II and U1 snRNP) to promote their parent gene expression in the nuclei. Long non-coding RNA (LncRNA) could also act as competitive endogenous RNAs to compete for microRNA binding.

Seeking biomarkers for diagnosis and prognosis of CNS diseases and to guide treatment is always one of top priorities in clinic research. As we know, the network of circRNAs, microRNAs, piRNA and lncRNAs needs to maintain a delicate dynamic balance to regulate cellular homeostasis, many of them have recently been studied extensively as biomarkers in CNS disorders. Once one of them is dysregulated, disease may occur (**Figure 1**) (Hentze and Preiss, 2013; Chen and Yang, 2015; Shen et al., 2015; Weiss et al., 2015). So measuring this network as a diagnostic marker and a possible marker for treatment monitoring is constantly promoted in recent studies. For example, circulating U2 small nuclear RNA fragments has been proposed to be a novel diagnostic biomarker for primary CNS lymphoma (Baraniskin et al., 2016). Long non-codingRNAs (lncRNA) and circulating miRNAs can be used as biomarkers for CNS disorders, such as lncRNA Sox2OT for AD and PD, lncRNA NEAT1 for Huntington’s disease (HD) (Wu et al., 2013), miR-451 for amyotrophic lateral sclerosis ALS (Grasso et al., 2014), miRNA 200 family for glioblastoma (Areeb et al., 2015), miR-326 for MS (Fenoglio et al., 2012) and serum miR-210 for cerebral ischemia and stroke (Ouyang et al., 2013). Piwi-interacting RNAs (piRNA) largely exert effects on transcription and genomic maintenance, expression of which in adult male spontaneous hypertensive rats were examined 24 h after focal ischemia. It was revealed that of ∼40,000 piRNAs analyzed, 105 piRNAs were significantly altered in cerebral cortex with 54 upregulated and 51 downregulated (Dharap et al., 2011). It was also observed that 4,527 of 15,849 total mouse circRNAs observed overlap with human circRNAs, and 11,322 of 15,849 were evolutionary conserved (Rybak-Wolf et al., 2015). These circRNAs are more stable than linear-RNAs, indicating circRNAs themselves could serve as biomarkers of CNS disorders (Qu et al., 2015).

Hypoxia additionally regulates back-splicing and generation of circRNAs. cZNF292 exhibits a proangiogenic function in endothelium (Boeckel et al., 2015) while the parental gene ZNF292s intron related with entorhinal cortical volume is associated with one AD-specific single-nucleotide polymorphism (SNP) (Furney et al., 2011).

Synapse dysfunction is a main contributor to CNS diseases (Dalkara and Alarcon-Martinez, 2015; Pasqualetti et al., 2015), whereas circRNAs are strongly enriched in synaptoneurosomes compared to the whole-brain lysate and cytoplasm (Rybak-Wolf et al., 2015). This inspires many to study the synaptoneurosomes-related aberrant circRNA released for CNS diseases diagnosis. Recent studies demonstrated aberrant circRNA expression in a disease condition-specific transcriptome analysis in blood cells themselves, or either active or passive release from diseased tissues (Memczak et al., 2015). Moreover, exosomes (<1000 nt) released by endothelial cells and neurons participate in angiogenic sprouting and support neuron activity, respectively (Chivet et al., 2013; Haqqani et al., 2013). Normally, the high stability of exo-circRNA (350 nt) (Li Y. et al., 2015) is due to the protection of exosomes or some specific sequence features or due to protein partner binding of circularing circRNAs (Li Y. et al., 2015). CNS diseases may compromise the BBB structure resulting in loss of small molecules such as microRNAs (18-23 nt) (Sun et al., 2013; Dalkara and Alarcon-Martinez, 2015) and microvesicles with an average diameter of 100 nm into blood or CSF, suggesting CNS circRNAs transporting out of the BBB (Faure et al., 2006). Overall, these data suggest a potential non-invasive tool to get CNS disease information from blood or CSF. Indeed, their abundance and specific expression patterns suggest that these molecules may have additional functions which remain to be discovered.

## Conclusion

Although circRNAs possess potential value as non-invasive clinical biomarkers for CNS disorders, a considerable amount of future work remain to be invested. It is important to accurately identify with high sensitivity which circRNAs are specifically dys-regulated in disease situations. For clinical application, the study population needs to be phenotypically well-defined. In addition, these circRNA biomarkers may be combined with other biomarkers and imaging tools to improve the power for diagnosis and prognosis. Furthermore, longitudinal understanding of the fundamental factors that influence miRNA expression such as age, environmental factors, and co-morbid conditions should also be considered.

## Author Contributions

Professor A-DX contributed the research concept and design. Moreover he finished critical revision of the article. Doctor DL finished the collection and assembly of data. Then she wrote the article. They all shared equal contribution in data analysis.

## Conflict of Interest Statement

The authors declare that the research was conducted in the absence of any commercial or financial relationships that could be construed as a potential conflict of interest.

The reviewer KC and handling Editor declared their shared affiliation, and the handling Editor states that the process nevertheless met the standards of a fair and objective review.
